# Individual and health facility factors and the risk for obstructed labour and its adverse outcomes in south-western Uganda

**DOI:** 10.1186/1471-2393-11-73

**Published:** 2011-10-14

**Authors:** Jerome K Kabakyenga, Per-Olof Östergren, Eleanor Turyakira, Peter K Mukasa, Karen Odberg Pettersson

**Affiliations:** 1 Division of Social Medicine and Global Health, Department of Clinical Sciences, Lund University, CRC, Entrance 72, 205 02 Malmo, Sweden; 2 Department of Community Health, Mbarara University of Science and Technology, Mbarara, Uganda; 3 EngenderHealth/Fistula Care Project, Kampala, Uganda

## Abstract

**Background:**

Obstructed labour is still a major cause of maternal morbidity and mortality and of adverse outcome for newborns in low-income countries. The aim of this study was to investigate the role of individual and health facility factors and the risk for obstructed labour and its adverse outcomes in south-western Uganda.

**Methods:**

A review was performed on 12,463 obstetric records for the year 2006 from six hospitals located in south-western Uganda and 11,180 women records were analysed. Multivariate logistic regression analyses were applied to control for probable confounders.

**Results:**

Prevalence of obstructed labour for the six hospitals was 10.5% and the main causes were cephalopelvic disproportion (63.3%), malpresentation or malposition (36.4%) and hydrocephalus (0.3%). The risk of obstructed labour was statistically significantly associated with being resident of a particular district [Isingiro] (AOR 1.39, 95% CI: 1.04-1.86), with nulliparous status (AOR 1.47, 95% CI: 1.22-1.78), having delivered once before (AOR 1.57, 95% CI: 1.30-1.91) and age group 15-19 years (AOR 1.21, 95% CI: 1.02-1.45). The risk for perinatal death as an adverse outcome was statistically significantly associated with districts other than five comprising the study area (AOR 2.85, 95% CI: 1.60-5.08) and grand multiparous status (AOR 1.89, 95% CI: 1.11-3.22). Women who lacked paid employment were at increased risk of obstructed labour. Perinatal mortality rate was 142/1000 total births in women with obstructed labour compared to 65/1000 total births in women without the condition. The odds of having maternal complications in women with obstructed labour were 8 times those without the condition. The case fatality rate for obstructed labour was 1.2%.

**Conclusions:**

Individual socio-demographic and health system factors are strongly associated with obstructed labour and its adverse outcome in south-western Uganda. Our study provides baseline information which may be used by policy makers and implementers to improve implementation of safe motherhood programmes.

## Background

Obstructed labour i.e. failure of descent of the fetal presenting part in spite of adequate uterine contractions [1,2] is a major cause of maternal morbidity and mortality in low income countries and accounts for approximately 8% of maternal deaths globally [3]. Studies in low-income countries have reported the prevalence of obstructed labour to be between 2% to 8% of all institutional deliveries [2,4,5]. This is most likely an underestimate as the majority of maternal deaths due to obstructed labour as a primary cause of death are rarely documented, but instead document the terminal cause of death, hence are classified as sepsis, ruptured uterus or haemorrhage rather than the underlying cause [6]. The main obstetric causes of obstructed labour in low income countries include cephalopelvic disproportion, malposition and malpresentation.

According to Thaddeus and Maine [7] who presented the "Three Delays Model", the chain of factors affecting the outcome of obstructed labour in low-income settings include both cultural and socio-economic factors. Adherence to traditional childbirth practices and individual beliefs as well as poverty restricting the family's ability to pay for transport is directly related to delay in seeking skilled care at birth (Delay I) but also in reaching the facility once the decision has been taken (Delay II). The association between being poor and limited use of health care services is well established although the direction of this association may differ from context to context [8,9]**.**Factors pertaining to the health care system interact in a complex manner, where one set of factors influence the others. The resources and skills may, for example, be available but for various reasons not offered in time (Delay III) consequently discouraging families from seeking care another time due to perceived low quality of care (Delay I) [7].

In most sub-Saharan countries including Uganda, women are traditionally expected to give birth at home [10] and consequently delay their health care seeking in childbirth, even if complications arise. Moreover, women are often marginalized in decision making regarding where and when to seek care [11]. Unofficial financial demands from health workers prevent women of badly needed maternal health services [10,12]. Inadequately developed health care systems including poor infrastructure, poor transportation and poor obstetric services are also major contributors to obstructed labour [4,13].

In a community study conducted in Gulu district in the northern part of Uganda, Orach [14] reported that obstructed labour was responsible for 26% of maternal deaths. However a national assessment of Uganda's obstetric care services in 2006 concluded that obstructed labour was responsible for 22% of all maternal deaths due to direct causes [15]. Through implementation of programmes such as the Sector Wide Approaches, strengthening family planning programs and increasing access to emergency obstetric care, Uganda has only managed to reduce maternal mortality from 557 maternal deaths per 100,000 live births in 1995 to 435 maternal deaths per 100,000 live births in 2006 [16,17]. It is therefore highly unlikely that Uganda will reduce maternal mortality to achieve the Millennium Development Goal (MDG) target of 131 maternal deaths per 100,000 live births by 2015 [18].

In order to increase the knowledge base for a more successful intervention against obstructed labour in low income settings, it is crucial to gain deeper insights in the roles of socio-economic factors and the health care system in the causal chain of the mentioned outcome. The aim of this study was to investigate the role of individual socio-demographic factors and factors related to the performance of the health system regarding the risk of obstructed labour and its adverse outcomes in a in a low-income sub-Saharan setting like south-western Uganda.

## Methods

### Study design and setting

A retrospective review of obstetric records was conducted in six hospitals namely Mbarara Regional Referral which also doubles as a university teaching hospital, Kitagata, Ishaka Adventist, Comboni, Ibanda, and Rushere Community located in five neighbouring districts of south-western Uganda. Table 1 shows the hospitals and the district of location, locality (urban/rural), category/ownership and deliveries for the year 2006. Kitagata, Ishaka Adventists and Comboni hospitals were at the time of conducting the study located in Bushenyi district (split into 5 districts since 1^st ^July, 2010). While the other 3 hospitals Mbarara, Ibanda, Rushere are located in Mbarara, Ibanda and Kiruhura districts respectively. Isingiro district did not have a hospital of its own and comprehensive emergency care services were sought from neighbouring Mbarara hospital.

**Table 1:** Hospitals in the study by district, location, category/ownership, and total deliveries for 2006

Hospital	District	Locality (Urban/Rural)	Category-Ownership	Total deliveries per hospital for 2006
Mbarara Regional referral	Mbarara	Urban	Regional Referral - Public	6164
Kitagata	**º**Bushenyi	Rural	General - Public	1339
Ishaka Adventist	**º**Bushenyi	Urban	General - *PNFP	1658
Comboni	**º**Bushenyi	Rural	General - *PNFP	792
Ibanda	Ibanda	Urban	General - *PNFP	1927
Rushere Community	Kiruhura	Rural	General - *PNFP	583

**º**Bushenyi was administratively split into 5 districts with effect from 1^st ^July 2010

*PNFP = Private Not For Profit

The districts of Mbarara, Bushenyi, Ibanda, Kiruhura, and Isingiro with a population of about one and a half million people share borders and have overall similar socio-economic and cultural conditions and use the local dialect of Runyankore as the common language.

The Uganda's health care system is structured in such way that there are corresponding health units or services at different levels of the administrative structure. The village health team (VHT) is the lowest level while a national referral hospital is the highest level of care [19,20] as shown in Table 2**.** Comprehensive emergency obstetric care services, especially operative delivery and blood transfusion, are available in all general, regional referral and national referral hospitals. According to our knowledge all the hospitals in the study were by structure able to offer a full range of comprehensive emergency obstetric care services at all times. Services offered in public hospital are officially free of charge although due to frequent shortages of drugs/supplies, patients/clients are requested to procure missing items from private pharmacies. Mbarara and Ibanda hospitals were the two hospitals with specialists (Obstetricians) while the other hospitals had general doctors as their highest ranked clinicians.

**Table 2:** Structure of Uganda national health system

Health unit	Services	Location	Population
Village Health Team	Outreach services	Village	1000
Health Centre (HC) II	Outpatient, antenatal care, immunization and outreach	Parish	5000
Health Centre III	HC II services plus inpatient care and environmental health	Sub-county	20,000
Health Centre IV	HC III services plus surgery and supervision of lower health units	County	100,000
General Hospital	General hospital care	District	500,000
Regional Referral Hospital	Specialists services	Region	2,000,000
National Referral Hospital	Advanced Tertiary care	National	27,000,000

Adapted from Government of Uganda Health Strategic Plan II, 2005/06-2009/10 [19] & Rutebemberwa et al., 2009 [20]

### Sample size and data collection

Twelve thousand four hundred and sixty three (12,463) obstetric records of women who were admitted in the maternity wards of the six hospitals (Mbarara, Kitagata, Ishaka, Comboni and Ibanda) from January 1 through December 31, 2006 were reviewed. The data collectors were midwives proficiently trained to collect data from women's obstetric files or charts and to validate the diagnosis of obstructed labour using admission, delivery and theatre registers. Data was recorded in case record forms developed by the researchers and pre-tested on 200 maternity records for the year 2007. The case record was modified to correct observed inconsistencies. The case record form was designed to collect data on socio-demographic variables, labour, delivery and post-delivery periods. Computer data entry was performed using Epidata (Epidata Association, Denmark).

### Diagnosis of obstructed labour

The criteria we used for diagnosing obstructed labour in this study was admission to a hospital with a pregnancy of a gestational age of 28 weeks or more and having a clinical diagnosis of obstructed labour in the patient chart or having an operative intervention (i.e. vaginal or abdominal) for failed progress of labour due to cephalopelvic disproportion, malpresentation or malposition. Women for whom the diagnosis of obstructed labour could not be ascertained were classified as non-obstructed labour and still included in the study sample.

### Exclusion criteria

Women who were admitted post-partum (n = 114) were excluded from the sample, as well as women whose gestational age was < 28 weeks upon admittance or were recorded as abortion (n = 482 women), and women who were discharged before delivery (n = 687 women). This reduced the number of records included in the sample from 12,463 to 11,180.

### Study variables

#### Outcome variables

*Obstructed labour was classified as*: "with obstructed labour" or "no obstructed labour"

*Cause of obstructed labour was classified as*: "cephalopelvic disproportion", "malpresentation" or "malposition" as stated in obstetric file or chart.

*Neonatal outcome was classified as: *"live birth" or "stillbirth"

*Maternal outcome was classified as: *"alive" or "died in hospital"

Maternal complications: coded as "Yes" (if a woman had at least one complication during labour or childbirth) otherwise coded "No"

*Perinatal mortality rate *was defined as "stillbirths and deaths in the first week per 1000 total births (live births plus stillbirths)".

#### Exposure variables and covariates

*Age of the woman was divided into 3 age groups*: 15-19, 20-29 and ≥30 years. The age range was 15 to 49 years. The age group 20-29 years was taken as the reference age group.

*Parity was classified into 4 groups*: "0" (nulliparous), "1", "2-4", "≥5". Parity 2-4 was taken as reference category, as it is considered to be the one with minimal risk of obstructed labour.

*Place of residence was defined as districts of residence, which were: *"Mbarara", "Bushenyi", "Ibanda", "Isingiro", "Kiruhura" and other districts (districts other than the ones specified). Bushenyi district was used as a reference district since at the time of the study it was the only district with 3 hospitals, thus providing more accessibility to emergency comprehensive services required in cases of obstructed labour.

*Occupation of mother was classified as: *"salaried employee", "subsistence farmer", "housewife". Salaried employee group was used as a reference group on the assumption that salaried women would be in the position to have financial resources to access health care services faster. In this study only occupation is used as a proxy for socio-economic status as most of the records were missing education information regarding levels attained by women.

*Health facility attended*: Comboni hospital, Ibanda hospital, Ishaka Adventist hospital, Kitagata hospital, Mbarara regional referral hospital, Rushere Community Hospital. Mbarara regional referral hospital being a university teaching hospital was expected to offer a high level of care and thus was therefore used as a reference hospital in this study.

### Statistical methods

Women seeking obstetric services in one hospital are expected to be more similar than women who visit other hospitals for the same service. Therefore SVY routines (Stata Version 10 Software) for handling correlated data were utilised to estimate proportions (%) and 95% confidence intervals of women who had obstructed labour and its outcomes (perinatal death and maternal complications). Crude Odds Ratios (COR), Adjusted Odds Ratios (AOR) and their 95% confidence intervals (CI) were calculated by means of multivariate logistic regression analysis taking into consideration the clustering of women at the hospital level. The mixed effects model was applied to determine the adjusted effects of age, parity, and residence on perinatal death and maternal complications among women who experienced obstructed labour assuming a random intercept. It was assumed that hospitals would have different intercepts due to differences in the level of care and some unmeasured health system factors. However the effect of the studied covariates was expected to be similar across hospitals. Cases with missing values were excluded from these analyses. Data was analysed using STATA version 9 (STATA Corporation, College Texas USA).

### Ethical approval

Ethical clearance was applied and granted from Uganda National Council of Science and Technology and from Lund University, Sweden.

Permission to access obstetric records was obtained from respective medical superintendents of the hospitals included in our study.

## Results

Table 3 shows the socio-demographic characteristics of 11180 women, districts of residence, and hospital they utilised. Sixty one per cent were aged between 20 and 29 years while 36% were nulliparous. The majority were residents of Mbarara (35%) and Bushenyi (31%) districts. Information about the occupation of the women was only possible to obtain for less than half of the sample due to incomplete registration at the health facilities. Among women whose occupations were recorded one fifth (20.6%) were salaried employees or working in the business sector, the remaining were farmers or housewives. Mbarara Regional Referral hospital attended to more than a half (51%) of the women in our sample. Comboni, Ishaka and Kitagata hospitals, all located in Bushenyi district, at the time of the study attended to 28% of the women. Ibanda and Rushere hospitals attended to 16.1% and 4.3% of the women respectively. The majority of women receiving child birth care from Comboni hospital (98.9%), Ishaka Adventist hospital (98.7%), Kitagata hospital (91.2%) and Rushere Community Hospital (92.7%) were resident in the same district where the hospital is located. Among women seeking childbirth care at Mbarara Regional Referral and Ibanda hospitals however, 45.5% and 33.5% respectively came from other districts.

**Table 3:** Socio-demographic characteristics of 11,180 women, their districts of residence, and hospitals they utilized

Characteristics	Number (n)	*Percent (%)
*Age group of women (years)*		
15-19	1933	17.5
20-29	6770	61.3
30-49	2336	21.2
Missing	[141]	
		
*Parity*		
0	3811	35.7
1	2155	20.2
2-4	3210	30.1
≥5	1492	14.0
Missing	[512]	
		
*Singleton/Multiple*		
Singleton	9696	95.6
Multiple	451	4.4
Missing	[1033]	
		
*Occupation of mother *		
Salaried employee/business	1062	20.6
Subsistence farmer	1215	23.5
Housewife	2886	55.9
Missing	[6017]	
		
Mbarara	3916	35.0
Bushenyi	3510	31.4
Ibanda	1045	9.3
Isingiro	936	8.4
Kiruhura	905	8.1
Other districts	868	7.8
		
*Name of health facility attended*		
Comboni hospital	636	5.7
Ibanda hospital	1803	16.1
Ishaka Adventist hospital	1435	12.8
Kitagata hospital	1098	9.8
Mbarara regional referral hospital	5731	51.3
Rushere Community hospital	477	4.3

* Valid percent is presented

There were 1170 cases of obstructed labour in a sample of 11,180 records analysed, amounting to a prevalence of obstructed labour of 10.5%. Ibanda and Mbarara Regional Referral hospitals registered the highest prevalence of obstructed labour at 12.5% and 12.4% respectively. The causes of obstructed labour were cephalopelvic disproportion (63.7%), malpresentation (19.8%) and malposition (16.2%). Three women (0.3%) had babies with hydrocephalus. Caesarean section delivery was performed on 91.1% of the women with obstructed labour. The other methods of delivery included laparotomy (6.3%), vacuum extraction (0.8%), symphysiotomy (0.5%), forceps (0.4%) and craniotomy (0.2%).

The fetal outcome was known for 10,127 (90.6%) of the 11,180 women records analysed. The perinatal mortality rate was 141 per 1000 total births (165/1167) for women who had obstructed labour and 65 per 1000 total births (586/8960) among women in the non-obstructed group (OR 2.31: 1.92-2.78). Overall the perinatal mortality rate was 74 per 1000 total births.

The risk of maternal complications was 10.8% (126/1170) in women with obstructed labour compared to 1.4% (145/10,010) in women without obstructed labour. Thus the odds of complications were 8 times higher (95% CI of odds ratio: 6.32-10.45) among women with obstructed labour compared to those without.

Maternal complications observed among women with obstructed labour were ruptured uterus (7.1%), puerperal sepsis (3.4%), bladder injury (1.8%), postpartum haemorrhage (1.2%), fistulae (1.4%) and one case of disseminated intravascular coagulation. While in the non-obstructed group the main complications were postpartum haemorrhage (0.5%), retained placenta (0.4%) and puerperal sepsis (0.1%). Thirteen women (1.2%) died as a result of obstructed labour while 38 women (0.4%) died in the non-obstructed labour group.

Table 4 shows the association between socio-demographic characteristics, district of residence, health facility and obstructed labour. The risk of obstructed labour in the age group of 15-19 years of age was 43% statistically significant higher than that of the reference age group of 20-29 years. Nulliparous women and women who had given birth once before had 52-53% higher statistical significant risk of obstructed labour than the reference parity group of 2-4, while in grand multiparous women the risk was 32% higher than the reference group. Women resident in Isingiro had a 37% statistically significant higher risk of getting obstructed labour than women from the reference district of Bushenyi. However women whose residences were located in the districts of Mbarara and Ibanda the risk of obstructed labour was 50% less than women from Bushenyi. Women who delivered in Comboni hospital showed a statistically significant higher risk of obstructed labour than those who delivered in the reference hospital of Mbarara Regional Referral i.e. the reference hospital (OR 2.98, 95% CI: 1.77-5.02). However women who delivered in Kitagata (OR 0.41, 95% CI: 0.31-0.54) and Rushere (OR 0.29, 95% CI: 0.10-0.47) hospitals showed a lower risk of obstructed labour than those who delivered from Mbarara.

**Table 4:** Association (OR, 95% CI) between socio-demographic characteristics, district of residence, health facility and obstructed labour

Characteristics	n	%	COR (95% CI)
*Age group of woman (years)*			
15-19	263	13.6	1.43 (1.23-1.67)
20-29	651	9.6	1.0 (ref)
≥30	237	10.1	1.07 (0.91-1.25)
			
*Parity*			
0	463	12.1	1.53 (1.30-1.80)
1	254	11.8	1.52 (1.26-1.82)
2-4	257	8.0	1.0 (ref)
≥5	152	10.2	1.32 (1.07-1.64)
			
*Singleton*			
Multiple	48	10.7	1.0 (ref)
Singleton	1,112	11.6	1.09 (0.80-1.48)
			
*Occupation of mother*			
Salaried employee/business	122	11.5	1.0 (ref)
Subsistence farmer	173	14.2	1.26 (0.98-1.61)
Housewife	354	12.3	1.08 (0.87-1.36)
			
*District of residence*			
Mbarara	376	9.6	0.57 (0.44-0.75)
Bushenyi	284	8.1	1.0 (ref)
Ibanda	99	9.5	0.57 (0.39-0.83)
Isingiro	190	20.3	1.37 (1.03-1.83)
Kiruhura	93	10.3	1.01 (0.72-1.43)
Other districts	128	14.7	1.04 (0.75-1.43)
			
*Health facility*			
Comboni hospital	70	11.0	2.98 (1.77-5.02)
Ibanda hospital	225	12.5	0.88 (0.67-1.14)
Ishaka Adventist hospital	87	6.1	1.01 (0.86-1.18)
Kitagata hospital	60	5.5	0.41 (0.31-0.54)
Mbarara regional referral hospital	709	12.4	1.0 (ref)
Rushere Community hospital	19	4.0	0.29 (0.10-0.47)

Table 5 shows the results of multivariate logistic regression analysis on the association between age, parity, district of residence and obstructed labour. Women who were in the age group15-19 years (AOR 1.21, 95% CI: 1.02-1.45), nulliparous (AOR 1.47, 95% CI: 1.22-1.78), women who had delivered once before (AOR 1.57, 95% CI: 1.30-1.91) and women resident in Isingiro district (AOR 1.40, 95% CI: 1.04-1.87) were at a statistically significant higher risk of obstructed labour. However women who were residents of Mbarara (AOR 0.58, 95% CI: 0.45-0.76) and Ibanda (AOR 0.62, 95% CI: 0.42-0.92) had a statistically significant lower risk of having obstructed labour.

**Table 5:** The association (OR, 95% CI) between age, parity, district of residence and obstructed labour: Result of multivariate logistic regression analysis

Women Characteristics	n = 10,586*	
	**COR (95% CI)**	**AOR (95% CI)**

*Age group of woman (years)*		
15-19	**1.41 (1.20-1.65)**	**1.21 (1.02-1.45)**
20-29	1.0 (ref)	1.0 (ref)
≥30	1.06 (0.90-1.24)	1.17 (0.95-1.43)
		
*Parity*		
0	**1.53 (1.30-1.80)**	**1.47 (1.22-1.78)**
1	**1.51 (1.25-1.82**)	**1.57 (1.30-1.91)**
2-4	1.0 (ref)	1.0 (ref)
≥5	1.32 (1.07-1.64)	1.15 (0.90-1.46)
		
*District of residence*		
Mbarara	**0.58 (0.45-0.76)**	**0.58 (0.45-0.76)**
Bushenyi	1.0 (ref)	1.0 (ref)
Ibanda	**0.60 (0.41-0.87)**	**0.62 (0.42-0.90)**
Isingiro	**1.39 (1.04-1.86)**	**1.40 (1.04-1.87)**
Kiruhura	1.02 (0.72-1.45)	1.05 (0.74-1.48)
Other districts	1.08 (0.78-1.49)	1.20 (0.79-1.52)

*Women with complete data on age, parity and district of residence

A subset of data from women who had obstructed labour was analysed to identify socio-demographic characteristics, which were associated with perinatal death (Table 6). In women with obstructed labour, the risk of perinatal death progressively increased with age and parity. Lack of paid employment and residing in the districts of Isingiro, and other districts outside the study area was linked to a higher risk for perinatal deaths. Babies born to women with obstructed labour in Ibanda hospital were a higher risk of perinatal death than babies born to women with obstructed labour in Mbarara regional referral hospital (the reference).

**Table 6:** The association (OR, 95% CI) between socio-demographic characteristics, occupation, district of residence, health facility and perinatal death

Characteristics	n/N	%	COR (95% CI)
*Age group of woman (years)*			
15-19	25/262	9.5	0.69 (0.43-1.12)
20-29	82/649	12.6	1.0 (ref)
≥30	52/237	21.9	1.93 (1.31-2.86)
			
*Parity*			
0	41/462	8.9	0.40 (0.26-0.63)
1	22/253	8.7	0.40 (0.23-0.68)
2-4	50/256	19.5	1.0 (ref)
≥5	46/152	30.3	1.81 (1.13-2.90)
			
*Singleton/multiple*			
Singleton	160/1119	14.3	1.40 (0.54-3.62)
Multiple	5/48	10.4	1.0 (ref)
			
*Occupation *			
Salaried employee/business	4/122	3.3	1.0 (ref)
Subsistence farmer	27/173	15.6	5.45 (1.85-16.02)
Housewife	42/353	11.9	3.98 (1.40-11.35)
			
*District of residence*			
Mbarara	36/375	9.6	0.82 (0.50-1.37)
Bushenyi	32/282	11.3	1.0 (ref)
Ibanda	15/99	15.2	1.39 (0.72-2.70)
Isingiro	34/190	17.9	1.70 (1.01-2.87)
Kiruhura	16/93	17.2	1.62 (0.84-3.12)
Other districts	32/128	25.0	2.60 (1.51-4.48)
			
*Health facility *			
Comboni hospital	8/70	11.4	0.85 (0.39-1.83)
Ibanda hospital	49/225	21.8	1.84 (1.25-2.70)
Ishaka Adventist hospital	8/86	9.3	0.68 (0.32-1.45)
Kitagata hospital	5/59	8.5	0.61 (0.24-1.57)
Mbarara regional referral hospital	93/708	13.1	1.0 (ref)
Rushere Community hospital	2/19	10.5	0.78 (0.18-3.43)

Multivariate logistic regression analysis was performed on a sub-population of women (n = 1113) with obstructed labour who had complete data on age, parity and district of residence to determine their independent impact on perinatal death (Table 7). The analysis reveals that grand multiparous status (AOR 1.89, 95% CI: 1.11-3.22) and women resident in other districts (AOR 2.85, 95% CI: 1.60-5.08) were at a statistically significant higher risk of having perinatal deaths as an adverse outcome. However women with obstructed labour who had never delivered before (AOR 0.40, 95% CI: 0.23-0.68) and those who had delivered one time (AOR 0.40, 95% CI: 0.23-0.70) had a statistically significant lower risk of having perinatal death as an adverse outcome.

**Table 7:** The association (OR, 95% CI) between age, parity, district of residence and perinatal death: Result of multivariate logistic regression analysis

Characteristics (n = 1113)	COR (95% CI)	AOR (95% CI)
*Age group (years)*		
15-19	0.68 (0.42-1.12)	1.02 (0.58-1.79)
20-29	1.0 (ref)	1.0 (ref)
≥30	1.94 (1.31-2.88)	0.91 (0.56-1.50)
		
*Parity*		
0	**0.41 (0.26-0.65)**	**0.40 (0.23-0.68)**
1	**0.39 (0.23-0.68)**	**0.40 (0.23-0.70)**
2-4	1.0 (ref)	1.0 (ref)
≥5	**1.76 (1.09-2.83)**	**1.89 (1.11-3.22)**
		
*District of residence*		
Mbarara	0.83 (0.49-1.40)	0.92 (0.54-1.58)
Bushenyi	1.0 (ref)	1.0 (ref)
Ibanda	1.41 (0.72-2.76)	1.39 (0.70-2.77)
Isingiro	1.61 (0.93-2.78)	1.62 (0.92-2.84)
Kiruhura	1.60 (0.81-3.15)	1.41 (0.70-2.83)
Other districts	**2.58 (1.47-4.51)**	**2.85 (1.60-5.08)**

The odds of perinatal death were about five and half times higher among women whose main occupation was of subsistence farming compared to women with salaried jobs or traders. Similarly housewives had 4 times higher odds of perinatal death compared to women with salaried income (see Table 6). However, the occupation variable could not be included in the multivariate model due to substantial missing data.

Partographs were found attached to 45.8% (536) of maternal records of women with obstructed labour. However only 19 (3.5%) partographs were satisfyingly documented; 13 in Comboni hospital, 4 in Mbarara regional referral hospital, 1 in Kitagata hospital and 1 in Rushere community hospital.

## Discussion

We determined the prevalence of obstructed labour in our sample to be 10.5%. Our findings showed that obstructed labour was associated with individual factors such as age, parity and socio-demographic factors such as occupation and place of residence and with particular health facility at which the woman gave birth. The association between low age, lower parity, district of residence and obstructed labour remained statistically significant even after multivariate regression analyses.

The proportion of women with obstructed labour who developed complications was 8 times that of those who did not have the condition. Thirteen women (1.2%) died from obstructed labour, compared to thirty eight (0.4%) in the non-obstructed labour group. We also found that adverse outcomes of obstructed labour, such as perinatal death, were associated with older age, high parity, occupation, district of residence and particular health facility. The association between old age, other districts and high risk of perinatal death remained strong after multivariate analysis.

The observed overall prevalence of obstructed labour of 10.5% for the six hospitals is high but, Mugerwa in an unpublished study reported a prevalence of 13% for Mbarara regional referral hospital for the years 1993 - 1995 while in our study the prevalence for the same hospital was 12.4%. To our knowledge, there are no previously published studies on the magnitude of obstructed labour in Uganda. However, studies conducted elsewhere in Africa have reported prevalence rates of between 0.9% - 7% [2,5,21,22].

The main obstetric causes of obstructed labour in our study were cephalopelvic disproportion, malpresentation and malposition which have also been reported by other authors elsewhere [2,4,21,22]. Cephalopelvic disproportion is mostly due to contracted pelvis and adolescent or early marriages. In south-western region of Uganda contracted pelvis could be attributed to malnutrition during childhood. Though rich in food crops, this region has consistently registered the highest levels of stunting among children in national demographic surveys conducted between 1995 and 2006 [23].

Women who are delivering for the first or second time may be young and as such be at a higher risk of obstructed labour than those who have delivered more times. Grand multiparous women may also be at a higher risk of obstructed labour from malposition and malpresentation. The finding that women whose district of residence was Isingiro had the highest risk for obstructed labour can be attributed to lack of a hospital which offers comprehensive obstetric services. Women diagnosed with obstructed labour therefore have to be transported on poor roads to Mbarara where they can access appropriate care. On the other hand, the low risk of obstructed labour for women resident in Mbarara and Ibanda districts found in our study could be related to the general good health services and transport infrastructure in the two districts. It should also be noted also that the two hospitals were the ones with resident Obstetricians.

Moreover, we found that adverse outcome of obstructed labour such as perinatal death was strongly associated with increasing parity, residence in other districts outside the study area. Women who have ever delivered may opt to deliver at home in subsequent pregnancies thereby exposing themselves to complications and a similar trend was reported in the Uganda demographic health survey of 2006 [23] where delivery by women in health facilities progressively decreased from 58% in nulliparous to 31% in those with parity 6 and above. In the same survey the south-western region was found to have only 31% of women delivering under the care of skilled birth attendants.

Our study showed that women whose occupation was subsistence farming or housewife were at a higher risk of having perinatal death as an adverse outcome compared to salaried women. Resource poor women may not be able to raise the money required in the event of an obstetric emergency and therefore face delay I and II as described by Thaddeus and Maine [7]**.**The association between low socio-economic status and obstructed labour has also been reported by other authors [24-26]. Obstructed labour, if not well managed in the early stages, leads to severe or debilitating complications and sometimes death [27]. In our sample the main maternal complications or adverse outcomes observed were ruptured uterus, puerperal sepsis, bladder injury, postpartum haemorrhage, fistulae and are similar to what has been reported by other authors [2,4,5,22].

The partograph, which is a low cost tool for monitoring labour, has been shown to reduce the occurrence of prolonged/obstructed labours [28]. The finding in our study that less than a half of all maternal records diagnosed with obstructed had partographs attached and that only 3.5% of these were completed may be an indication of poor monitoring of labour and child birth in the hospitals studied. A study on assessment of partograph use during labour conducted in Rukungiri district (neighbouring Bushenyi district) reported a 70% use but with only 2% of the partographs fulfilled the standard monitoring of fetal heart rate [29]. Studies conducted elsewhere in Africa have reported poor partograph utilization in lower health facilities [30,31] and a high utilization rate in a tertiary hospital [32].

Our study findings point to the effect of socio-economic factors and the health system structure in the causal chain of obstructed labour. Since 2000 Uganda government through the Health Sector Strategic Plans [19] has been in a process of increasing availability and accessibility of emergency obstetric services in the whole country. One of the key interventions was upgrading of health centre level IV to offer comprehensive emergency obstetric services. It was hoped that since health centre IV are in easy reach of most of the population they would help to manage most obstetric emergencies. A study in 2003 by Mbonye et al. [15] indicated that only 13% and 9% of health centre IV could offer blood transfusion and caesarean section services respectively. Although there are no published studies on the current availability of emergency obstetric services, anecdotal information indicates that most health centre IVs in the country are not able to offer a full range of emergency obstetric services. This is evidenced by the MDG report, which shows Uganda's poor performance towards attaining the target of reducing maternal mortality by the year 2015 [18].

Our study provides baseline information on the individual socio-economic and health system factors associated with obstructed labour and can be used by policy makers and implementers to improve on management of obstructed labour in south-western Uganda.

### Methodological considerations

Since the sample comprised all pregnant women who delivered in the six hospitals during the study period, selection bias is very unlikely. Women sought obstetric care across the border of their district of residence, which most likely depended on their judgement where care was most accessible. Thus the sample could be considered a "pragmatic catchment area" of the six investigated hospitals, and as such reasonably representative on a more generalized level. However since occupation, the proxy indicator we used for socio-economic position was registered for about half the sample, analyses, which included this variable could well be sensitive to selection bias either way.

Regarding dependent misclassification that is whether different hospitals were likely to systematically under (or over) classifying obstructed labour is highly unlikely. Since the study relied on clinical notes in the records there could have been inaccuracies in the diagnosis, which were missed. This also makes dependent misclassification regarding the outcomes and individual socio-demographic variables unlikely. However the diagnosis and management of obstructed labour like most clinical conditions is not clear cut. Some authors who include Ali [5] in Sudan, Nwogu-Ikoju [4] in Nigeria, Gessessew [2] in Ethiopia, Chhabra et al. [33] in India have included vacuum, forceps and symphysiotomy as intervention methods in addition to caesarean section, destructive operations for obstructed labour. However Melah et al. [21] and Ozumba & Uchegbu [22] from Nigeria, Adhikari et al. [34] from India reported caesarean sections or destructive operations as the only interventions applied in cases of obstructed labour. In our study we found that some hospitals had applied the mentioned interventions to achieve vaginal delivery in a few cases of obstructed labour. van Roosmalen [35,36] advocates for an increase in use of symphysiotomy in management of obstructed labour low resource settings. This method may be preferable to caesarean section, which may jeopardize the future obstetric life of women who have poor access to emergency obstetric care.

Potential confounding between the main exposures variables were handled by means of a multivariate logistic regression analysis where all variables were controlled for each other.

## Conclusions

Socio-demographic and health system factors are strongly associated with obstructed labour and its outcome in south-western Uganda. Our study provides baseline information on the individual socio-economic and health system factors associated with obstructed labour. The findings can be used by policy makers and implementers to improve on management of obstructed labour in the country as a whole. In order to address the problem of obstructed labour, interventions are needed both within the community and the health system level. Further research in the management of obstructed labour within health facilities is advocated for and it also pertinent to study the community's perception of prolonged/obstructed labour and actions taken when it occurs.

## Competing interests

The authors declare that they have no competing interests.

## Authors' contributions

JK participated in planning, conceptualising, data collection and writing the draft manuscript. P-OO & KOP participated in planning, conceptualizing, designing and study implementation. ET participated in designing the data collection protocol, organising field activities, data collection, data entry and running the analysis. PK participated in the designing of the data collection protocol form data collection, and verifying all the clinical records. All authors participated in interpreting data, reviewed the manuscript.

All authors read and approved the final manuscript

## Authors' Information

JK is a PhD student at Lund University, Sweden and a Lecturer of Public Health at Mbarara University of Science and Technology (MUST), Uganda. KOP is a Researcher/Lecturer of Social Medicine & Global Health at Lund University, Sweden.

P-OO is a Professor of Social Medicine at Lund University, Sweden. ET is Lecturer of Biostatistics/Epidemiology at MUST. PM is a Senior Medical Associate with EngenderHealth/Fistula Care Project, Kampala, Uganda and at the time of conducting the study, PM was a Lecturer of Obstetrics & Gynaecology at MUST.

## Pre-publication history

The pre-publication history for this paper can be accessed here:

http://www.biomedcentral.com/1471-2393/11/73/prepub
